# Temporal Trends in the Burden of Major Depressive Disorder and Dysthymia in Asia, 1990–2023: Findings from the Global Burden of Disease Study 2023

**DOI:** 10.3390/healthcare14213563

**Published:** 2026-10-22

**Authors:** Shenyan Wang, Qunhao Zhou, Chaofan Li, Yongqi Lin, Mingmei Cheng, Baoliang Zhang, Lu Liu, Lidan Liu, Xiaofeng Liu, Hongxing Kan, Yanzhong Wang, Wanqing Xie

**Affiliations:** 1 Department of Intelligent Medical Engineering, School of Biomedical Engineering, Anhui Medical University, Hefei 230032, China; 2 Research Center for Translational Medicine, The Second Affiliated Hospital of Anhui Medical University, Hefei 230601, China; 3 School of Mental Health and Psychological Sciences, Anhui Medical University, Hefei 230032, China; 4 School of Life Course and Population Sciences, Faculty of Life Science and Medicine, King’s College London, London SE1 1UL, UK; 5 Department of Radiology and Biomedical Imaging, Yale University, New Haven, CT 06520, USA; 6 School of Medical Informatics Engineering, Anhui University of Chinese Medicine, Hefei 230012, China; 7 Center for Xin’an Medicine and Modernization of Traditional Chinese Medicine of IHM, Anhui University of Chinese Medicine, Hefei 230012, China; 8 Department of Burns, The First Affiliated Hospital of Anhui Medical University, Hefei 230022, China

**Keywords:** burden of disease, dysthymia, depressive disorders, major depressive disorder, risk factors

## Abstract

**Highlights:**

**What are the main findings?**

- MDD ASIR showed marked short-term fluctuations during 2019–2023, with a non-significant increase followed by a significant decline, whereas dysthymia remained relatively stable.
- Within the GBD 2023 framework, intimate partner violence (IPV)-attributable MDD burden was estimated only among females and, for the first time in 2023, accounted for the largest proportion of MDD-related DALYs among the three risk factors examined in Asia.

**What are the implications of the main findings?**

- Mental health systems in Asia may benefit from a dual-track strategy: maintaining flexible response capacity for short-term fluctuations in MDD burden while ensuring sustained, long-term care for chronic dysthymia.
- The findings highlight the need for targeted prevention and mental health support for women experiencing intimate partner violence within women’s health frameworks across Asia.

**Abstract:**

**Background:** This study aims to comprehensively evaluate the burden of depressive disorders and their subtypes (major depressive disorder (MDD) and dysthymia) in Asia, with particular attention to periods overlapping with the COVID-19 pandemic. **Methods:** We examined the burden of depressive disorders, including the numbers and age-standardized rates (ASRs) of incidence, prevalence, and disability-adjusted life years (DALYs) across Asian nations. We also assessed the proportion of MDD-related DALYs attributable to three risk factors within the GBD 2023 framework. Stratified analyses were conducted by sex, age, disease subtype, and geographic location. Estimated annual percentage changes (EAPCs) were calculated to examine temporal trends. **Results:** From 1990 to 2023, the burden of MDD in Asia consistently exceeded that of dysthymia. China and the Democratic People’s Republic of Korea showed relatively limited ASDR fluctuations across 2019–2021 and 2021–2023 compared with several other Asian countries and territories. As for dysthymia, it showed relatively stable trends in ASRs across Asian nations during 1990–2023. However, India showed the largest increases in ASIR, ASPR, and ASDR (EAPCs: 0.30, 0.36, and 0.38, respectively), far exceeding those of the second-ranking country. In 2023, MDD-related DALY rates peaked among individuals aged 15–19 years, whereas dysthymia-related DALY rates peaked among those aged 65–69 years. Additionally, in 2023, IPV accounted for the largest proportion of MDD-related DALYs among the three risk factors examined in Asian females (8.64%). **Conclusions:** The depressive disorder burden in Asia showed distinct temporal patterns, with more pronounced short-term fluctuations in MDD and relative stability in dysthymia, highlighting the potential value of a dual-track mental health strategy that combines flexible response capacity for MDD with sustained, long-term care for chronic dysthymia.

## 1. Introduction

Depressive disorders represent a major global public health challenge, contributing substantially to both the global disease and economic burden [1,2,3,4]. Asia accounts for over half of the global burden of depressive disorders and is undergoing rapid population ageing amid profound demographic and economic transitions [5,6,7]. A more effective response to this challenge requires that research move beyond estimating the overall burden and explicitly differentiate between the two principal subtypes: major depressive disorder (MDD) and dysthymia [8]. MDD is characterized by an episodic course, whereas dysthymia is marked by more persistent depressive symptoms and a chronic course, and these differences may be reflected in distinct epidemiological trajectories [9,10]. Moreover, persistent dysthymic symptoms may represent a vulnerability state potentially associated with an increased risk of subsequent MDD episodes. Therefore, distinguishing between MDD and dysthymia is critical for characterizing their respective temporal, geographical, age-specific, and sex-specific patterns of disease burden in Asia.

Although existing research has advanced our understanding of the burden of depressive disorders, several critical gaps remain that necessitate further investigation. Tian et al. systematically assessed the burden of depressive disorders and their subtypes (MDD and dysthymia) in China and its provinces from 1990 to 2021 [11]. Nevertheless, their study provided limited analysis of dysthymia, and its scope was restricted to a single country. Chen et al. used data from the Global Burden of Disease (GBD) 2019 to examine temporal trends in mental disorders across Asian countries [6]. However, this analysis did not differentiate between MDD and dysthymia or characterize the temporal changes in the burden of depressive disorders and their subtypes during periods overlapping with the COVID-19 pandemic. To address these gaps and inform the development of more targeted prevention and control strategies, a comprehensive analysis of the temporal trends in the disease burden of depressive disorders and their subtypes is imperative. Leveraging data from the GBD 2023, our study comprehensively examined the burden of depressive disorders, MDD, and dysthymia separately. We quantified incidence, prevalence, and disability-adjusted life years (DALYs) by sex and age group across Asian countries and territories from 1990 to 2023. To characterize temporal changes in disease burden during periods overlapping with the COVID-19 pandemic, we stratified the analysis into three distinct periods: 1990–2019, 2019–2021, and 2021–2023, consistent with the timing of pandemic emergence and the subsequent post-pandemic period reported in previous studies [12,13]. Furthermore, we estimated the DALYs of MDD attributable to specific risk factors within the GBD 2023 framework.

## 2. Materials and Methods

### 2.1. Overview

The GBD 2023 database estimated the burden of 298 causes of death, 380 diseases and injuries, and 88 risk factors, across 204 countries and territories [8]. The data included measures of incidence, prevalence, mortality, years of life lost (YLLs), years lived with disability (YLDs), and DALYs from 1990 to 2023. Data for this study were obtained through the Global Health Data Exchange (GHDx) online query tool (http://ghdx.healthdata.org/gbd-results-tool, accessed on 13 October 2025), accessed in October 2025. Population data by country or territory, age, and sex were obtained from the World Population Prospects 2024 of the United Nations Department of Economic and Social Affairs, Population Division [14]. Asia was defined according to the geographic classification of the United Nations M49 standard (Standard Country or Area Codes for Statistical Use) [15]. According to this classification, Asia comprises 50 countries or areas. Based on the availability of data in the GBD 2023 database, Hong Kong Special Administrative Region and Macao Special Administrative Region were excluded because corresponding GBD 2023 estimates were unavailable. Taiwan was included because GBD 2023 provided corresponding estimates. Therefore, the present study included 49 countries and areas in Asia [15]. Detailed information on the data sources, indicators, and downloaded datasets used in this study is provided in Supplementary File S1 Tables S1–S4.

This study presents a comprehensive assessment of the incidence, prevalence, and DALYs of overall depressive disorders and their subtypes—MDD and dysthymia—using data from the GBD 2023. Our analysis is stratified by sex and age groups across Asian countries and territories. To characterize temporal changes, we examined the data across three predefined intervals: 1990–2019, 2019–2021, and 2021–2023. The latter two intervals were selected to capture short-term changes potentially associated with the COVID-19 pandemic and the subsequent period. Moreover, the study describes the burden of MDD attributable to specific risk factors at the national and regional levels in Asia.

### 2.2. Definition of Depressive Disorders and Description of Burden

In GBD 2023, depressive disorders were categorized into two categories according to the DSM-5-TR or ICD-10 criteria, including MDD (DSM-5-TR: 296.20-26, 296.30-36; ICD-10: F32.0-9, F33.0-9) and dysthymia (DSM-5-TR: 300.4, ICD-10: F34.1) [16,17,18]. MDD is defined as an episodic mood disorder characterized by one or more major depressive episodes that fulfil the diagnostic criteria. Dysthymia is defined as a chronic depression with symptoms milder than MDD but with a longer duration. The term “dysthymia” refers to the GBD 2023 cause category and should not be considered equivalent to persistent depressive disorder. Case definitions were consistently applied within the GBD 2023 framework throughout the 1990–2023 study period, ensuring temporal comparability of the estimates.

### 2.3. Incidence, Prevalence, and Disability-Adjusted Life Years

Incidence refers to the number of new cases of a given disease during a given period in a specified population, whereas prevalence refers to the total number of cases of a given cause in a specified population at a designated time [19]. DALYs consist of two components: YLLs and YLDs. YLLs account for the age and sex at the time of death, thus evaluating the health loss attributed to early mortality. YLDs focus on the loss of functional health due to diseases or injuries, estimating the number of years lived with health conditions by considering both the severity and duration of the disease [8,19]. In this study, incidence, prevalence and DALYs were obtained from the GBD 2023 study and represent modelled estimates rather than directly observed or clinically diagnosed cases. To facilitate comparisons across populations with different age structures, age-standardized rates were used in this study. The age-standardized incidence rate (ASIR), age-standardized prevalence rate (ASPR), and age-standardized DALY rate (ASDR) represent the incidence, prevalence, and DALY burden adjusted to a standard age distribution, thereby reducing the influence of demographic differences and enabling more meaningful comparisons across countries and over time.

### 2.4. Risk Factors

This study investigated the contribution of specific risk factors to the burden of MDD. Although depressive disorders include both MDD and dysthymia, the attributable burden estimates available in the current GBD 2023 framework do not include dysthymia. Accordingly, the risk factor analysis was limited to MDD, for which attributable burden estimates were available for IPV, CSA, and BV. Our analysis focused on MDD-related DALYs attributable to these risk factors, with further stratification at the level of Asian countries and territories to describe geographical variations in attributable burden.

### 2.5. Statistical Analyses

To assess the temporal trends in age-standardized rates (ASR) of incidence, prevalence, and DALYs for depressive disorders, the estimated annual percentage change (EAPC) was calculated. ASR, expressed per 100,000 population, was computed using the following formula:

$$\mathrm{ASR}=\left(\frac{\sum_{i=1}^{A}a_{i}w_{i}}{\sum_{i=1}^{A}w_{i}}\right)\times 100,000$$

where *a_i_* is the age-specific rate for the *i*-th age group, *w_i_* is the number of individuals in the *i*-th age group in the standard population, and *A* is the total number of age groups. For Asia as a whole, ASR were calculated using the WHO 2000–2025 World Standard Population, with age-specific population weights obtained from the SEER standard population dataset, whereas ASR for individual countries and areas were obtained directly from the GBD 2023 database [20]. Given the different age-standardization references, the ASR for Asia was used to describe the overall burden and temporal patterns in Asia, while country-level ASRs were used for comparisons among individual countries and areas. Direct numerical comparisons between the ASR for Asia and country-level ASRs were not performed. The EAPC was derived from a regression model characterizing the trend in ASR over a specified period. The model was specified as *ln*(*ASR*) = *α* + *β*X + *ε*, where *Y* = *ln*(*ASR*), *X* is the calendar year, *α* is the intercept, *β* is the slope or trend, and *ε* is the error term. The EAPC was then calculated as 100 × [*exp*(*β*) − 1], representing the annual percentage change.

The EAPC and its corresponding 95% confidence interval (CI) were estimated from the linear regression model. An increasing trend in ASR was defined as an EAPC greater than zero with the lower bound of its 95% CI also greater than zero, whereas a decreasing trend was defined as an EAPC less than zero with the upper bound of its 95% CI less than zero. If neither condition was met, the trend was considered statistically stable, indicating that a statistically significant increase or decrease could not be established. All estimates of incidence, prevalence, DALYs, and age-standardized rates were presented with 95% uncertainty intervals (UIs) obtained directly from the GBD 2023 database. These 95% UIs reflect the uncertainty derived from the GBD estimation process and were used to describe the statistical uncertainty of disease burden estimates. The statistical analyses were performed using the R software (version 4.2.1).

## 3. Results

### 3.1. Temporal Trends and Epidemiological Characteristics of Depressive Disorders

**Figure 1:**
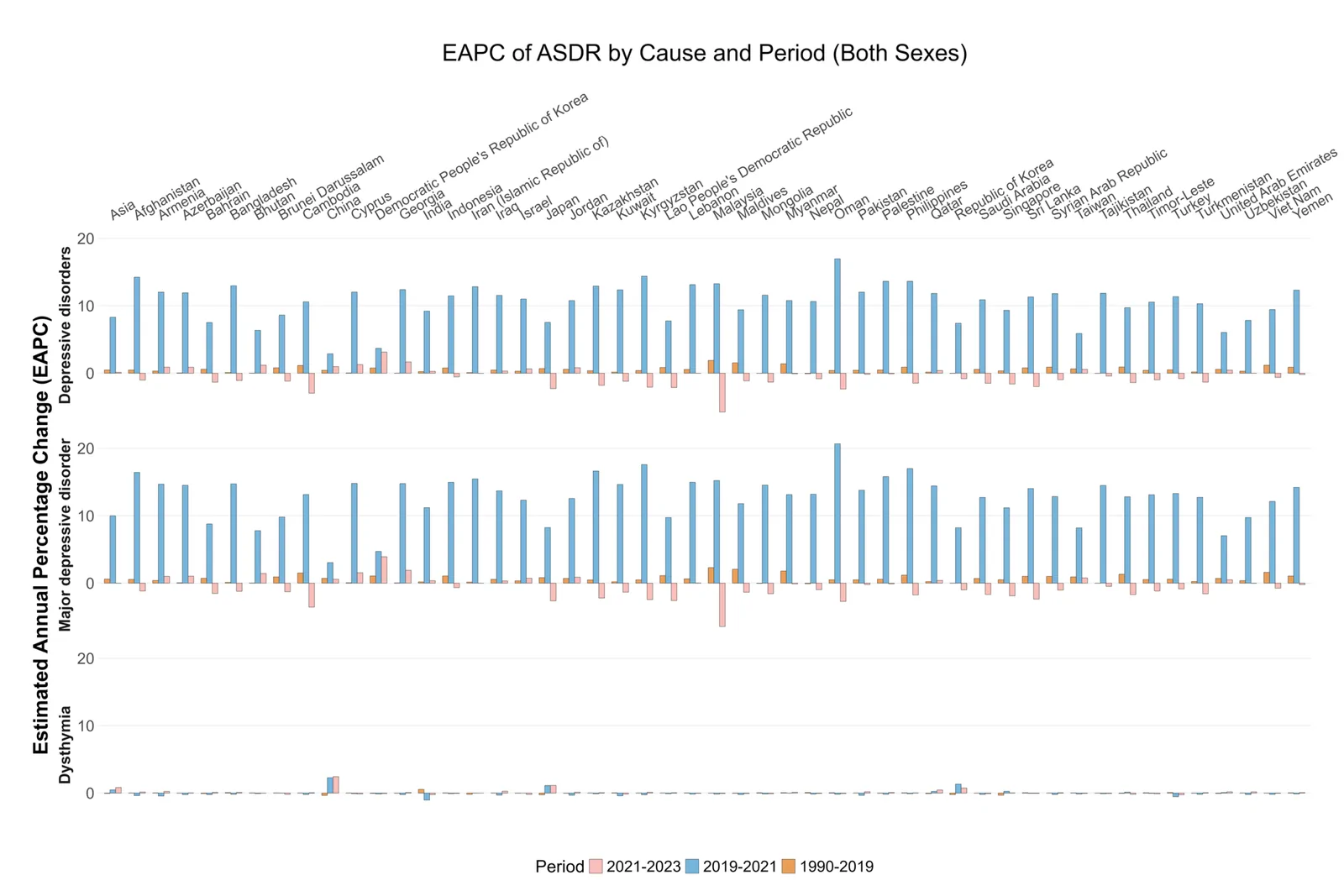
Estimated annual percentage changes (EAPCs) of age-standardized disability-adjusted life year rates for depressive disorders and their subtypes (major depressive disorder and dysthymia) in Asia by nations and periods. Values represent EAPCs (%); 95% confidence intervals (CIs) are not displayed.

In 2023, depressive disorders continued to represent a major contributor to the disease burden in Asia, manifesting with substantial incidence, prevalence, and DALYs across the region. Depressive disorders accounted for approximately 166.86 million incident cases in Asia, corresponding to an ASIR of 3333.24 per 100,000 population (95%UI: 2907.23 to 3817.32). The number of prevalent cases reached 185.44 million, with an ASPR of 3604.84 per 100,000 persons (95%UI: 3086.53 to 4258.99). The associated DALYs totaled 31.65 million, yielding an ASDR of 623.47 per 100,000 persons (95%UI: 423.32 to 891.79). From 1990 to 2019, the burden of depressive disorders in Asia remained relatively stable, followed by a substantial increase in the point estimates of ASIR, ASPR, and ASDR during 2019–2021; however, none of these increases reached statistical significance Notably, ASIR declined sharply between 2021 and 2023 (EAPC: −16.02 [95%CI:−16.08 to −15.96]), while ASPR and ASDR demonstrated statistically stable (EAPCs: 0.21 [95%CI:−0.74 to 1.17] and 0.13 [95%CI:−1.17 to 1.44], respectively) (Figure 1, Table 1, Supplementary File S1: Figures S6–S8).

**Figure 2:**
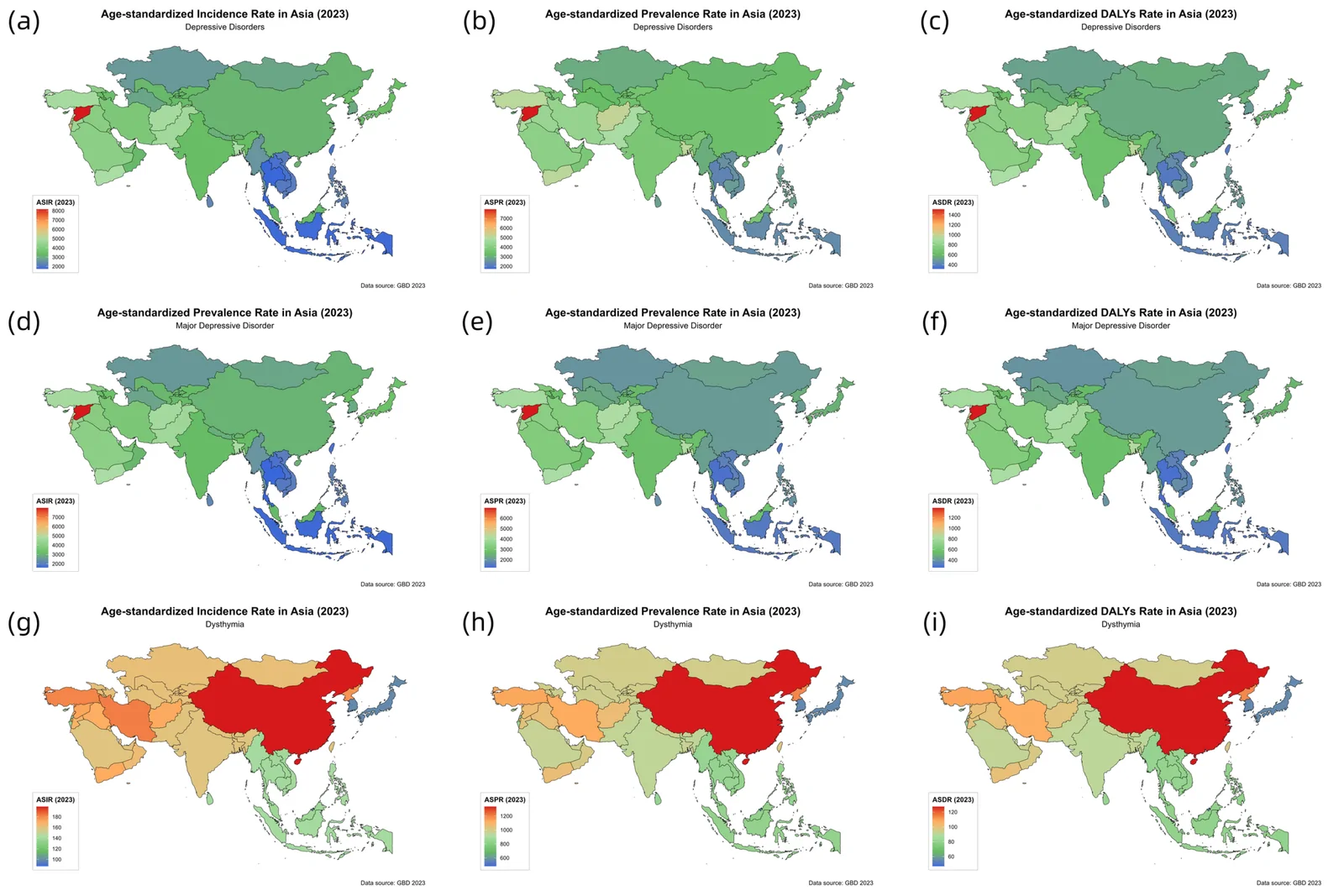
Age-standardized rates (ASRs) of incidence (ASIR), prevalence (ASPR), and DALYs (ASDR) per 100,000 population for depressive disorders and their subtypes (major depressive disorder and dysthymia) across Asian countries and territories in 2023. (**a**) Depressive disorders ASIR; (**b**) depressive disorders ASPR; (**c**) depressive disorders ASDR; (**d**) major depressive disorder ASIR; (**e**) major depressive disorder ASPR; (**f**) major depressive disorder ASDR; (**g**) dysthymia ASIR; (**h**) dysthymia ASPR; (**i**) dysthymia ASDR. Data source: Global Burden of Disease Study 2023 [21].

China and India, as two of the most populous countries in Asia, had the largest absolute numbers of individuals affected by depressive disorders across all three indicators. These large numbers were consistent with their substantial population sizes, with the number of affected individuals in 2023 approximately five times higher than that observed in Pakistan, the third-ranked country in Asia. In 2023, the Syrian Arab Republic had the highest ASIR, ASPR, and ASDR (8160.89 [95%UI: 6805.34 to 9551.32], 7951. 42 [95%UI: 6220.2 to 10,049.91], and 1482.84 [95%UI: 967.03 to 2189.28] per 100,000 persons, respectively) among all Asian countries and territories, each being nearly 1.5 times higher than the corresponding values in the country or territory with the second-highest rates. Compared with many other Asian countries and territories, China and the Democratic People’s Republic of Korea showed relatively low EAPC point estimates for ASDR during 2019–2021 (EAPCs: 2.88 [95% CI: −2.77 to 8.85] and 3.70 [95% CI: −2.30 to 10.07], respectively); however, the corresponding 95% CIs crossed zero, indicating that these short-term changes were not statistically significant. During 2021–2023, both countries showed upward trends in ASDR, with EAPCs of 0.99 (95% CI: 0.64 to 1.35) and 3.14 (95% CI: 0.52 to 5.84), respectively. Overall, unlike the sharp increases followed by substantial declines observed in several other Asian countries and territories, China and the Democratic People’s Republic of Korea displayed relatively limited fluctuations in ASDR across these two periods. By contrast, Oman showed the most pronounced rise in the EAPC for ASIR between 2019 and 2021 yet was among the countries with the sharpest declines from 2021 to 2023. Over the entire period 1990–2023, Malaysia exhibited the greatest overall increase in the EAPCs of ASIR, ASPR, and ASDR for depressive disorders across all countries and territories (Figure 1 and Figure 2, Supplementary File S1: Figures S1, S2 and S4–S52, and Tables S6–S8).

**Figure 3:**
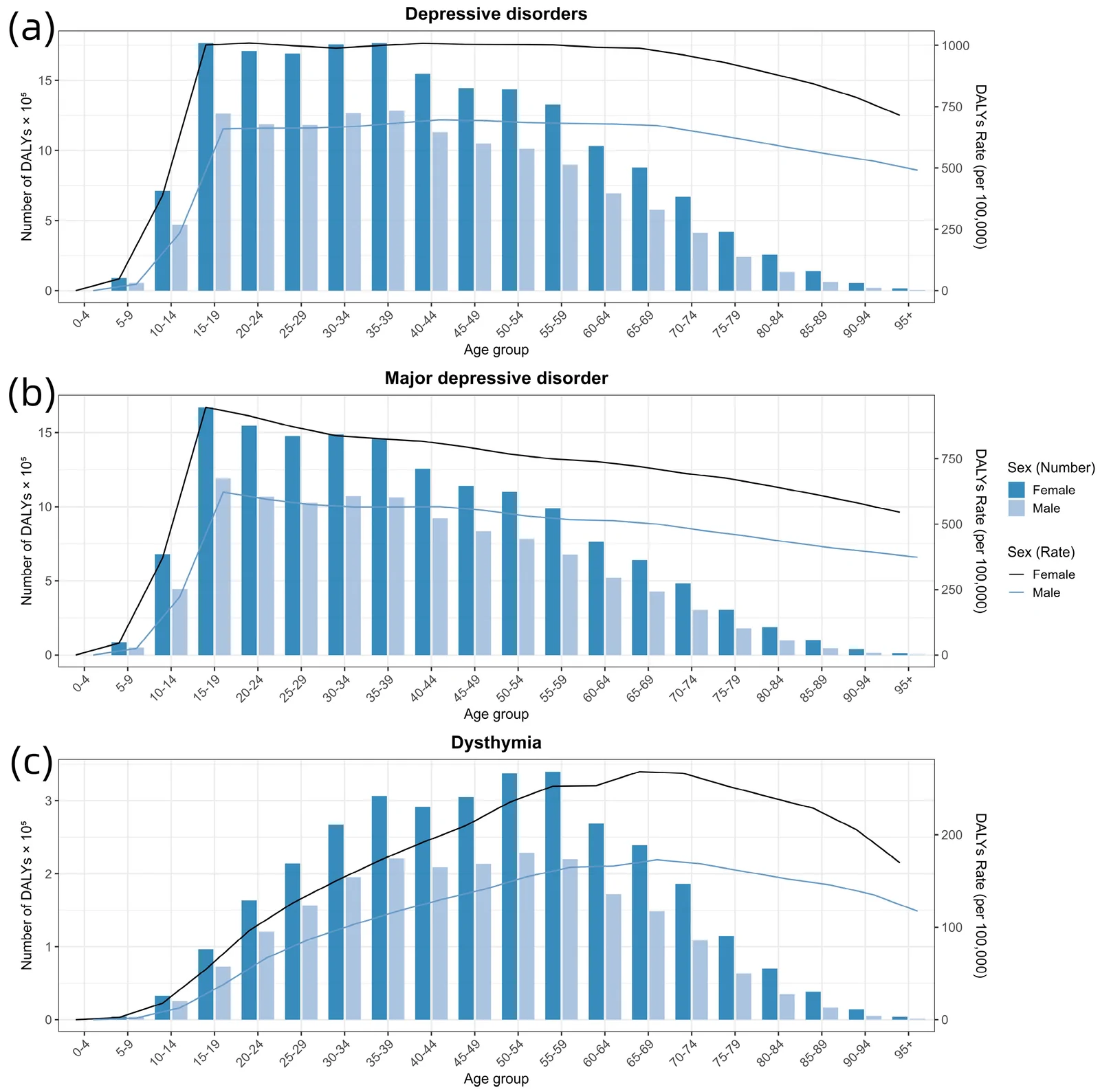
Disability-adjusted life years (DALYs) of depressive disorders and their subtypes (major depressive disorder and dysthymia) in Asia by age groups and sex in 2023; Bars represent the number of DALYs (×10^5^), while lines represent the corresponding DALY rates per 100,000 population. No 95% uncertainty intervals (UIs) are displayed. (**a**) depressive disorders; (**b**) major depressive disorder; (**c**) dysthymia.

Females consistently exhibited a higher burden of depressive disorders than males across all age groups and indicators. In 2023, Asian incidence rate peaked in the 15–19 age group for both sexes, while prevalence rate was highest in the 65–69 age group. The highest DALY rates were observed in the 20–24 age group for females and in the 40–44 age group for males (Figure 3, Supplementary File S1: Figures S53 and S54 and Table S15).

### 3.2. Burden and Distinct Patterns of Depressive Subtypes

MDD constituted the predominant portion of the depressive disorders burden in Asia. In 2023, there were 157.93 million incident cases of MDD, with an ASIR of 3166.05 per 100,000 persons (95%UI: 2742.14 to 3643.71). The number of prevalent cases reached 129.60 million, with an ASPR of 2594.45 per 100,000 persons (95%UI: 2109.64 to 3229.32). MDD accounted for 26.14 million DALYs, with an ASDR of 523.55 per 100,000 persons (95%UI: 349.32 to 754.83). The temporal trends of MDD were closely similar to those observed for overall depressive disorders (Figure 1, Table 1, Supplementary File S1: Figures S9–S11).

In contrast, dysthymia accounted for a substantially smaller proportion of the depressive disorders’ burden. Incident cases of dysthymia totaled 8.93 million, with an ASIR of 167.18 per 100,000 persons (95%UI: 136.370 to 205.86). Prevalent cases amounted to 57.82 million, with an ASPR of 1047.26 per 100,000 persons (95%UI: 876.15 to 1228.46). Dysthymia led to 5.51 million DALYs, with an ASDR of 99.92 per 100,000 persons (95%UI: 66.63 to 144.49). From 1990 to 2023, the ASIR, ASPR, and ASDR of dysthymia in Asia showed negligible change, with EAPCs of −0.03 (95%CI: −0.08 to 0.03), −0.1 (95%CI: −0.19 to 0), and −0.09 (95%CI: −0.18 to 0.01), respectively (Figure 1, Table 1, Supplementary File S1: Figures S12–S14).

The burden of MDD and dysthymia showed distinct cross-national variations in Asia. The number of individuals with MDD and dysthymia was highest in China and India, consistent with the overall depressive disorder distribution. In 2023, the Syrian Arab Republic had the highest estimated MDD ASIR, ASPR, and ASDR, reaching 7987.64 (95%UI: 6638.06 to 9376.08), 6982.95 (95%UI: 5301.90 to 9180.10), and 1382.15 (95%UI: 900.74 to 2040.01) per 100,000 population, respectively. By contrast, dysthymia showed its highest burden in China, with corresponding values of 198.98 (95%UI: 161.87 to 243.41), 1333.75 (95%UI: 1126.39 to 1518.86), and 127.3 (95%UI: 86.41 to 183.39) per 100,000 population in 2023 (Figure 2, Supplementary File S1: Tables S9–S14).

The temporal trends of MDD were generally consistent with those of overall depressive disorders. From 2019 to 2021, China and the Democratic People’s Republic of Korea showed the lowest EAPC point estimates for ASIR, ASPR, and ASDR. However, during 2021–2023, they recorded the smallest declines in ASIR, ranking first and second in terms of the least decrease. The Democratic People’s Republic of Korea also exhibited the greatest increases in ASPR and ASDR, whereas China showed statistically stable trends in both indicators. In contrast, Oman recorded the largest increase during 2019–2021 but this sharp upward pattern was not sustained in the subsequent period, whereas Malaysia showed the highest overall increases across 1990–2023 (Figure 1, Supplementary File S1: Figures S1, S2 and S4–S52, and Tables S9–S11).

Temporal changes in dysthymia burden were modest across all Asian nations, with EAPCs close to zero during 1990–2023. Although the overall trend remained stable, India experienced the largest increases in ASIR, ASPR, and ASDR over this period, with EAPCs of 0.3 (95%CI:0.21 to 0.40), 0.36 (95%CI:0.25 to 0.48), and 0.38 (95%CI:0.27 to 0.49), substantially exceeding those of the second-ranking country (all below 0.1 for the three indicators). In contrast, China, Singapore, the Republic of Korea, and Japan showed notable declines in ASIR, ASPR, and ASDR between 1990 and 2023, with China exhibiting the steepest decreases, with EAPCs of −0.21 (95%CI:−0.35 to −0.07), −0.34 (95%CI:−0.56 to −0.12), and −0.33 (95%CI:−0.55 to −0.11), respectively. Relative to other countries; Turkmenistan showed the largest decline in ASDR during 2019–2021, with an EAPC of −0.54 (95% CI: −0.94 to −0.13). (Figure 1, Supplementary File S1: Figures S1, S2 and S4–S52, and Tables S12–S14).

Age- and sex-related burden patterns were also distinct for MDD and dysthymia. For MDD, both sexes exhibited peak incidence, prevalence, and DALY rates in the 15–19 age group. In contrast, for dysthymia, the incidence rates for both males and females peaked in the 55–59 age group. The prevalence rates were highest in the 70–74 age group for females and the 65–69 age group for males, while the DALY rates peaked in the 65–69 age group for both sexes. Similar to overall depressive disorders, females consistently exhibited a higher burden than males across all age groups for both MDD and dysthymia (Figure 3, Supplementary File S1: Figures S53 and S54 and Tables S16 and S17).

### 3.3. Attributable Burden of Major Depressive Disorder by Psychosocial Risk Factors

**Figure 4:**
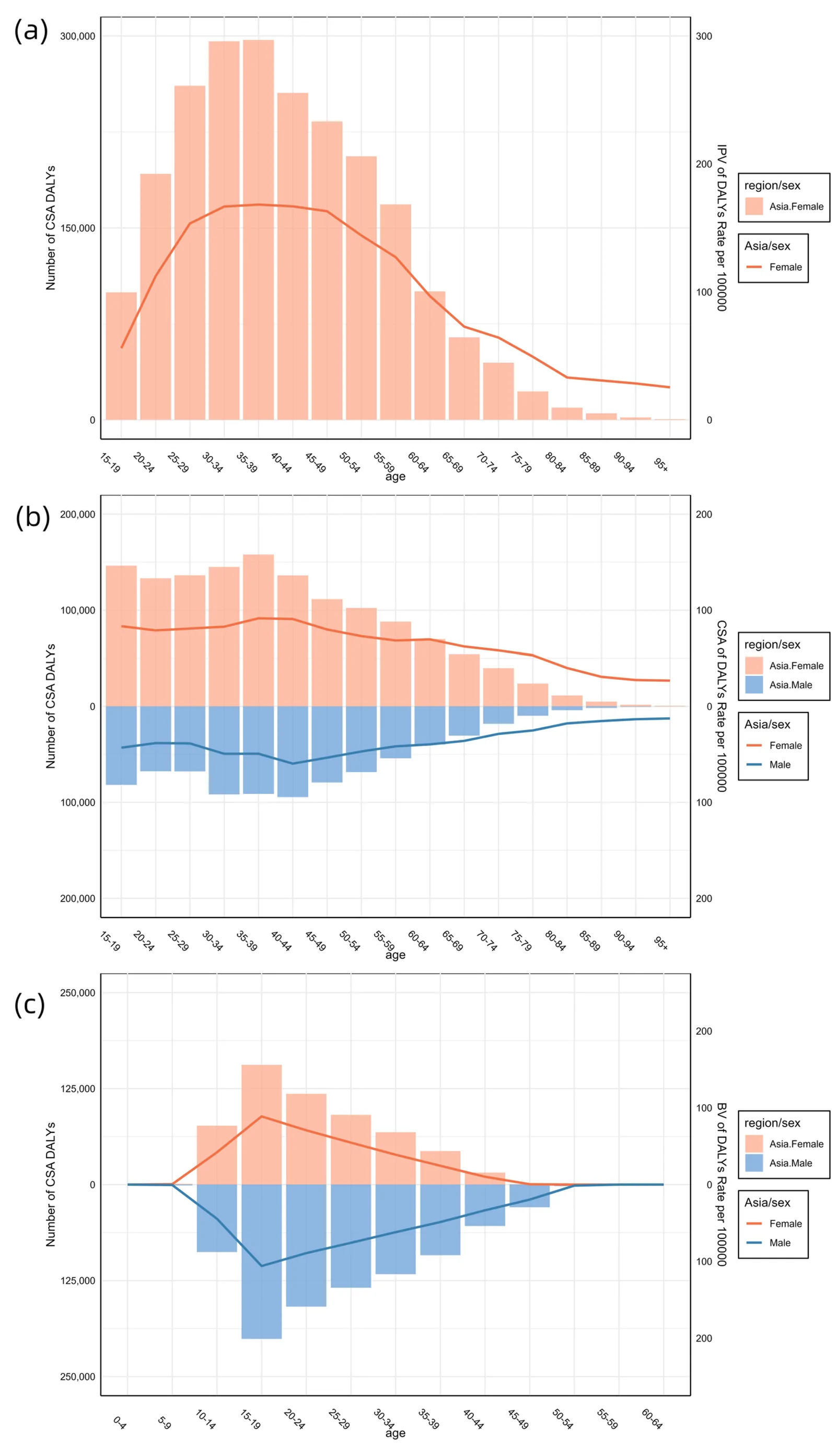
Disability-adjusted life years (DALYs) attributable to intimate partner violence (IPV), childhood sexual abuse (CSA), and bullying victimization (BV) for major depressive disorder (MDD) in Asia, by age groups and sex; Bars represent the number of attributable DALYs, and lines represent the corresponding DALY rates per 100,000 population. No 95% uncertainty intervals (UIs) are displayed. (**a**) intimate partner violence (IPV); (**b**) childhood sexual abuse (CSA); (**c**) bullying victimization (BV).

In 2023, IPV accounted for the largest proportion of MDD-related DALYs among the three risk factors examined (8.64%). CSA and BV, in contrast, showed decreasing attributable proportions from 1990 to 2023. Specifically, since 2019, the burden attributable to CSA declined from 9.91% to 8.28%, and that of BV dropped from 6.75% to 5.54%. In 2023, among psychosocial risk factors for MDD in Asia, IPV, CSA, and BV had ASDRs of 43.7 (95%UI: 17.07 to 79.19), 42.73 (95%UI: 4.28 to 102.72), and 31.42 (95%UI: 14.28 to 56.86) per 100,000 population. For IPV, MDD-related DALYs attributable to IPV were estimated only among females in the GBD 2023 framework, with the highest burden observed in the 35–39 age group. For CSA, the peak occurred at ages 40–44 in males and 35–39 in females. BV-related burden peaked in adolescents aged 15–19 in both sexes (Figure 4, Supplementary File S1: Figures S55–S57, and Tables S18–S20).

The attributable burden of MDD associated with IPV, CSA, and BV showed marked geographic heterogeneity across Asia. The highest proportions of MDD-related DALYs attributable to IPV were observed in Turkey (14.15%), Bangladesh (11.75%), Taiwan (Province of China) (10.59%), and Mongolia (10.55%); for CSA, the corresponding proportions were highest in India (10.45%), Timor-Leste (10.28%), the Republic of Korea (9.91%), and Japan (9.83%); for BV, they were highest in Qatar (14.20%), Brunei Darussalam (11.52%), Maldives (11.50%), and Jordan (10.97%). (Figure 4, Supplementary File S1: Figures S55–S57, and Tables S15–S17).

## 4. Discussion

This study systematically analyzed the disease burden and epidemiological characteristics of depressive disorders and their major subtypes—MDD and dysthymia—across Asian countries and territories from 1990 to 2023, using GBD 2023 data. A foremost observation of this study is the distinct patterns of MDD and dysthymia during the periods overlapping with the COVID-19 pandemic in Asia. Our detailed subtype analysis showed that the point estimates of incidence, prevalence, and DALYs for MDD increased substantially between 2019 and 2021, coinciding with the COVID-19 pandemic and associated societal disruptions, social isolation, and economic uncertainties. This increase was followed by a sharp decline in the MDD ASIR between 2021 and 2023, coinciding with the period of gradual mitigation of the COVID-19 pandemic. In contrast, the burden of dysthymia remained relatively stable across these periods. These findings suggest that MDD and dysthymia may exhibit distinct temporal patterns during large-scale societal crises such as the COVID-19 pandemic [22,23]. However, these observed patterns should not be interpreted as evidence of a causal effect of the COVID-19 pandemic, as they may also reflect changes in healthcare access, reporting practices, diagnostic ascertainment, model inputs, and other contextual factors. Furthermore, potential diagnostic overlap or misclassification between dysthymia and MDD in clinical and research settings may affect the observed epidemiological patterns of dysthymia, particularly during public health emergencies when greater attention may be directed toward acute depressive symptoms [24]. Consequently, enhancing diagnostic accuracy through improved clinical training, the systematic application of structured diagnostic tools, and heightened awareness of chronic depressive symptoms may facilitate the effective recognition of dysthymia and the provision of targeted interventions and comprehensive care.

The burden of MDD across Asian countries and territories showed pronounced differences. Notably, China and the Democratic People’s Republic of Korea showed relatively limited fluctuations in ASDR across 2019–2021 and 2021–2023. This pattern may partly reflect their early and stringent containment measures, which helped limit COVID-19 transmission and may have reduced some of the broader societal disruptions associated with the pandemic [25,26]. In contrast, Oman exhibited the largest increase in depressive burden from 2019 to 2021, but a marked decline from 2021 to 2023. This fluctuation may be linked to limitations in the health system, structural inequalities affecting migrants, and economic pressures [27]. Limited access to mental health services during lockdowns may have exacerbated psychological distress, particularly among migrant populations facing structural barriers. Meanwhile, rising unemployment, sociocultural expectations of “breadwinners,” and family-related stress may have intensified economic pressures. Social isolation and reduced physical activity may have further compounded mental health challenges [28]. The subsequent decline after 2021 may have reflected improvements in public health response, increased access to healthcare, and progress in telemedicine for mental health in Oman [29]. In other nations, the elevated burden of depression may be explained by distinct factors. For instance, the Syrian Arab Republic had the highest estimated MDD ASIR, ASPR, and ASDR, which may partly reflect ongoing conflict, economic instability, and fragile health infrastructure [30]. Notably, Malaysia showed the highest overall increases in ASIR, ASPR, and ASDR between 1990 and 2023. This rise may partly reflect persistent structural weaknesses in its mental health system, including a shortage of psychiatrists and limited specialized institutions. Sociocultural barriers, such as stigma and low public awareness of mental disorders, may have further restricted the development and utilization of mental health services [31]. Collectively, these MDD-specific findings suggest the importance of context-sensitive approaches to addressing depressive disorders across Asia. Given MDD’s high sensitivity to societal stressors, countries with fragile health systems or ongoing conflicts may require greater capacity to respond to acute mental health needs during periods of societal stress. Meanwhile, in some nations where the primary challenge stems from long-term deficits in healthcare infrastructure, sustained investments in the mental health workforce and community-based interventions, alongside stigma reduction campaigns, may help improve access to mental health care. Mental health systems across Asia should be sufficiently resilient to absorb societal shocks and manage surges in treatment demand during crises.

Dysthymia exhibited different temporal trends compared with MDD in Asia. Dysthymia demonstrated relative stability, with EAPCs remaining close to zero from 1990 to 2023. However, considerable heterogeneity was observed across countries. India showed the largest increase from 1990 to 2023, which may be attributable to limited and unevenly distributed mental health resources, gradual improvements in case detection, and broader sociocultural factors, including social stress and religious beliefs [32,33,34]. For example, recent initiatives such as the Systematic Medical Appraisal, Referral, and Treatment (SMART) mental health digital intervention in rural India have demonstrated the potential of digital and community-based approaches to improve the identification and management of common mental disorders. Such improvements in detection and service accessibility may have contributed, at least partly, to the observed increase in estimated disease burden [35]. By contrast, the burden of dysthymia declined in China, Singapore, the Republic of Korea, and Japan from 1990 to 2023, which may reflect the cumulative effects of improved mental health infrastructure, enhanced public awareness, and socioeconomic development [33,36]. Nevertheless, from 2019 to 2021, the estimated dysthymia burden in China, Japan, and the Republic of Korea showed some indication of an upward trend. This indication suggests that, despite long-term progress, dysthymia burden may still be influenced by acute societal stressors, potentially due to social isolation, economic insecurity, experiences of illness and bereavement, and disruptions in mental health services [11,37]. Meanwhile, some countries and territories showed declines in dysthymia burden over the same period, including Turkmenistan and Turkey. In Turkey, this decline may partly reflect reduced case detection associated with restrictions on mental health services and changes in healthcare accessibility during the pandemic [38]. Overall, the burden of dysthymia in Asia remained relatively stable from 1990 to 2023, but this finding should be interpreted cautiously. The apparent stability may partly reflect potential under-recognition of dysthymia, as its chronic but relatively mild symptom profile may reduce clinical attention and help-seeking, particularly in settings where mental health stigma remains prevalent [5]. In addition, acute social stressors may be associated with short-term changes in dysthymia burden, even against a backdrop of long-term stability. Therefore, public health strategies for dysthymia should be tailored to its chronic yet stress-sensitive nature. This may include the development of sustainable, long-term management systems, ideally integrated into primary care.

Age- and sex-specific patterns warrant careful consideration. Consistent with previous studies, females consistently exhibited a higher burden of both MDD and dysthymia across all age groups, a disparity may reflect a combination of biological factors, such as hormonal fluctuations, and psychosocial stressors, including gender-based inequality [6,39,40]. Notably, our findings showed that incidence, prevalence, and DALY rates of MDD all peaking in adolescence (15–19 age group). Early-onset MDD has been associated with an increased risk of recurrence and long-term negative impacts on social and occupational functioning [41]. In contrast, dysthymia exerts a more substantial burden among middle-aged and older adults, with DALY rates peaking at ages 65–69 years. This pattern may be associated with chronic physical conditions, changes in social roles, and the cumulative effects of persistent low mood [42,43]. Notably, the peak age of dysthymia burden in Asia is higher than the global average, which may be closely associated with the region’s pronounced population aging [21]. The accumulation of chronic physical conditions, social isolation, and insufficient long-term mental health care among older adults may collectively contribute to the persistent and prolonged course of dysthymia in this region [44]. These age- and sex-specific patterns suggest that greater attention may be warranted for female adolescents and older women in public health planning, with a focus on improved mental health literacy, early screening, and enhanced access to appropriate services.

Finally, our study highlights the contribution of psychosocial risk factors to the burden of MDD. Notably, although the GBD 2023 framework estimated IPV-attributable MDD burden only among females, IPV accounted for the largest proportion of MDD-related DALYs among the three risk factors examined by 2023, surpassing both CSA and BV. This shift may be associated with heightened stress, disruption of social and protective networks, loss of income, and decreased access to support and essential services, as suggested by previous research [45,46]. However, these factors were not directly examined in the present study, and the observed change in IPV-attributable MDD-related DALYs should therefore be interpreted cautiously rather than as evidence of a direct association with these factors. Meanwhile, CSA and BV also continued to contribute to MDD-related DALYs. The observed sex-specific pattern in CSA-attributable burden, with a higher burden among females, may relate to differential biological stress responses [47]. In China, the burden attributable to CSA has gradually declined in recent years, possibly reflecting the long-term effects of child protection and school-based education initiatives introduced to prevent abuse and sexual violence [48]. The burden of BV may be underestimated, partly because victims are often reluctant to disclose their experiences, underscoring the need for early identification and intervention [49,50]. Prioritizing the early identification and mitigation of these important risk factors through community-based education and early intervention programs may help reduce their potential long-term psychological consequences and improve mental health outcomes.

## 5. Limitations

Our study also has some limitations. First, limited mental health data coverage, particularly in low- and middle-income countries, may affect the accuracy of GBD estimates, as limited healthcare and diagnostic capacity may result in missed or misdiagnosed cases of depressive disorders and contribute to underestimation of the true burden. Conversely, variations in healthcare systems and reporting practices across countries may also contribute to potential overestimation of the burden in some settings. Consequently, it is difficult to determine whether the observed changes reflect true epidemiological changes in depressive disorders or are related to changes in detection or GBD modelling.

Second, the assessment of depressive disorders relies primarily on symptom reports and scale-based tools rather than objective biological markers, which may introduce measurement errors. Moreover, the widely used ICD and DSM diagnostic criteria, largely developed in high-income countries, may lack cultural sensitivity across diverse populations, and the establishment of more culturally adaptable diagnostic frameworks may be necessary in the future [51].

Third, although the 2019–2021 and 2021–2023 intervals were examined to characterize recent fluctuations coinciding with the COVID-19 timeline, no formal causal inference was conducted to quantify the independent effect of the pandemic on depressive disorder burden. Furthermore, each interval comprised only three annual data points, which may constrain the statistical robustness of the EAPC estimates and limit their interpretation as sustained temporal trends. Additionally, substantial variations in the timing and intensity of the pandemic, public health restrictions, and societal responses across Asian countries and territories hamper direct cross-country comparisons. Thus, observed temporal shifts should be strictly interpreted as descriptive patterns rather than as effects directly attributable to the pandemic.

Fourth, as a secondary analysis based on aggregate GBD data, our study inherently employs an ecological study design. Consequently, the observed associations at the population or national level cannot be directly inferred as individual-level causal relationships, which carries the risk of ecological fallacy.

Fifth, the risk factor analysis was limited to IPV, CSA, and BV because attributable burden estimates for MDD were available for these three risk factors within the GBD 2023 framework. Future research should investigate additional risk factors for MDD and explore the risk factors associated with dysthymia.

Finally, as our analysis relied on national-level GBD 2023 estimates. A more detailed sub-national analysis could provide valuable insights into regional variations in healthcare access and socio-economic factors, which may influence the burden of depressive disorders. However, due to limited data at this level, such analyses were beyond the scope of this study. Future research should focus on sub-national data to better capture these differences. Despite these limitations, our study provides the latest estimates of the burden of depressive disorders and their attributable risk factors, offering a scientific foundation for the formulation of mental health policies in Asia.

## 6. Conclusions

This study comprehensively delineates the Asian national burden of depressive disorders and their distinct subtypes, MDD and dysthymia, with a particular focus on their temporal trends observed during periods overlapping with the COVID-19 pandemic and the contribution of specific risk factors to MDD-related burden. MDD showed more pronounced short-term fluctuations during periods overlapping with the COVID-19 pandemic, whereas dysthymia remained relatively stable over the long term. The burden of both subtypes varied substantially across countries and territories and showed distinct age- and sex-specific patterns. Our findings highlight the importance of subtype-specific, context-sensitive, and age- and sex-tailored public health strategies to address current mental health needs and enhance healthcare system resilience against future public health challenges.

## Figures and Tables

**Table 1:** Incidence, prevalence, and disability-adjusted life years of depressive disorders and their subtypes in Asia: disease burden and temporal trends with 95% uncertainty and confidence intervals.

Location	1990		2019		2021		2023		EAPC, %, in ASRs from 1990 to 2023 (95% CI)	EAPC, %, in ASRs from 1990 to 2019 (95% CI)	EAPC, %, in ASRs from 2019 to 2021 (95% CI)	EAPC, %, in ASRs from 2021 to 2023 (95% CI)
	Number × 10^3^ (95%UI)	ASR (95%UI)	Number × 10^3^ (95%UI)	ASR (95%UI)	Number × 10^3^ (95%UI)	ASR (95%UI)	Number × 10^3^ (95%UI)	ASR (95%UI)				
Incidence												
Depressive disorders												
Asia	88,472.70 (75,062.89, 102,089.36)	2914.37 (2529.38, 3320.96)	160,726.90 (141,646.34, 178,274.57)	3358.16 (2956.63, 3734.37)	229,422.76 (201,717.30, 254,817.79)	4726.49 (4161.72, 5256.52)	166,856.49 (145,718.95, 190,771.73)	3333.24 (2907.23, 3817.32)	0.87 (0.6, 1.14)	0.51 (0.47, 0.56)	18.64 (−3.19, 45.39)	−16.02 (−16.08, −15.96)
Major depressive disorder												
Asia	83,638.61 (70,380.92, 97,511.28)	2747.59 (2366.96, 3161.41)	152,433.58 (133,192.73, 169,872.28)	3193.42 (2781.44, 3569.28)	220,825.35 (193,173.27, 245,394.98)	4560.84 (3994.9, 5073.5)	157,930.29 (136,542.91, 181,304.70)	3166.05 (2742.14, 3643.71)	0.92 (0.63, 1.2)	0.54 (0.49, 0.59)	19.51 (−3.35, 47.77)	−16.68 (−16.69, −16.67)
Dysthymia												
Asia	4834.09 (3928.14, 5936.33)	166.77 (136.23, 204.08)	8293.32 (6699.03, 10,229.78)	164.74 (134.15, 201.57)	8597.41 (6958.11, 10,608.94)	165.65 (134.55, 202.92)	8926.20 (7212.87, 11,041.22)	167.18 (136.37, 205.86)	−0.03 (−0.08, 0.03)	0.01 (−0.06, 0.07)	0.27 (−0.32, 0.87)	0.46 (−0.06, 0.99)
Prevalence												
Depressive disorders												
Asia	83,446.60 (68,588.49, 99,986.89)	2868.41 (2369.48, 3407.9)	153,141.84 (132,971.89, 179,069.38)	3126.72 (2705.05, 3660.95)	179,620.12 (155,370.00, 211,103.58)	3589.8 (3100.23, 4223.81)	185,437.10 (159,248.70, 218,926.74)	3604.84 (3086.53, 4258.99)	0.59 (0.47, 0.71)	0.36 (0.34, 0.39)	7.15 (−0.65, 15.56)	0.21 (−0.74, 1.17)
Major depressive disorder												
Asia	55,430.08 (43,064.90, 70,935.73)	1844.89 (1425.45, 2342.88)	102,497.28 (83,440.68, 127,753.60)	2138.46 (1734.72, 2659.64)	126,637.11 (103,953.00, 157,545.93)	2595.71 (2122.85, 3221.96)	129,598.43 (105,308.29, 161,525.99)	2594.45 (2109.64, 3229.32)	0.94 (0.76, 1.13)	0.59 (0.54, 0.65)	10.17 (−1.55, 23.29)	−0.02 (−1.7, 1.68)
Dysthymia												
Asia	28,728.34 (24,058.87, 33,664.12)	1049.97 (879.7, 1226.18)	52,154.74 (43,423.43, 61,548.67)	1018.16 (855.66, 1197.12)	54,844.26 (45,587.04, 64,509.80)	1030.07 (866.05, 1208.91)	57,815.22 (47,696.41, 68,212.39)	1047.26 (876.15, 1228.46)	−0.1 (−0.19, 0)	−0.06 (−0.17, 0.06)	0.58 (−0.41, 1.58)	0.83 (−0.04, 1.71)
DALYs (Disability-Adjusted Life Years)												
Depressive disorders												
Asia	14,049.29 (9312.50, 20,147.85)	471.23 (312.9, 672.69)	25,749.97 (17,681.53, 36,852.95)	530.16 (363.68, 757.82)	30,793.94 (21,086.22, 43,929.51)	621.89 (425.24, 888.42)	31,650.99 (21,528.27, 45,026.70)	623.47 (423.32, 891.79)	0.76 (0.61, 0.9)	0.48 (0.44, 0.51)	8.31 (−1.13, 18.64)	0.13 (−1.17, 1.44)
Major depressive disorder												
Asia	11,290.08 (7323.84, 16,658.34)	371.4 (241.32, 543.62)	20,758.46 (14,112.90, 30,225.68)	432.82 (295.44, 629.02)	25,560.57 (17,239.64, 36,908.62)	523.62 (355,755.92)	26,141.78 (17,497.48, 37,722.95)	523.55 (349.32, 754.83)	0.96 (0.77, 1.14)	0.61 (0.55, 0.67)	9.99 (−1.63, 22.98)	−0.01 (−1.69, 1.71)
Dysthymia												
Asia	2759.21 (1849.92, 3989.26)	99.84 (66.82, 143.57)	4991.51 (3322.13, 7217.21)	97.35 (65.29, 140.17)	5233.38 (3487.31, 7597.76)	98.27 (65.85, 141.46)	5509.21 (3663.93, 8036.73)	99.92 (66.63, 144.49)	−0.09 (−0.18, 0.01)	−0.05 (−0.16, 0.07)	0.48 (−0.53, 1.5)	0.83 (0.02, 1.65)

## Data Availability

Data used in this study were obtained from the Global Health Data Exchange Global Burden of Disease Results Tool (https://ghdx.healthdata.org/gbd-results-tool, accessed on 13 October 2025).

## Supplementary Materials

The following supporting information can be downloaded at https://www.mdpi.com/article/10.3390/healthcare14213563/s1 . Figure S1: Incidence; Figure S2: Prevalence; Figure S3: Asia; Figure S4: Afghanistan; Figure S5: Armenia; Figure S6: Azerbaijan; Figure S7: Bahrain; Figure S8: Bangladesh; Figure S9: Bhutan; Figure S10: Brunei_Darussalam; Figure S11: Cambodia; Figure S12: China; Figure S13: Cyprus; Figure S14: Democratic_People_s_Republic_of_Korea; Figure S15: Georgia; Figure S16: India; Figure S17: Indonesia; Figure S18: Iran__Islamic_Republic_of_; Figure S19: Iraq; Figure S20: Israel; Figure S21: Japan; Figure S22: Jordan; Figure S23: Kazakhstan; Figure S24: Kuwait; Figure S25: Kyrgyzstan; Figure S26: Lao_People_s_Democratic_Republic; Figure S27: Lebanon; Figure S28: Malaysia; Figure S29: Maldives; Figure S30: Mongolia; Figure S31: Myanmar; Figure S32: Nepal; Figure S33: Oman; Figure S34: Pakistan; Figure S35: Palestine; Figure S36: Philippines; Figure S37: Qatar; Figure S38: Republic_of_Korea; Figure S39: Saudi_Arabia; Figure S40: Singapore; Figure S41: Sri_Lanka; Figure S42: Syrian_Arab_Republic; Figure S43: Taiwan__Province_of_China_; Figure S44: Tajikistan; Figure S45: Thailand; Figure S46: Timor_Leste; Figure S47: Turkey; Figure S48: Turkmenistan; Figure S49: United_Arab_Emirates; Figure S50: Uzbekistan; Figure S51: Viet Nam; Figure S52: Yemen; Figure S53: Incidence; Figure S54: Prevalence; Figure S55: Intimate partner violence; Figure S56: Childhood sexual abuse; Figure S57: Bullying victimization; Table S1: GBD 2023 Disease Burden Data Extraction and Selection Criteria; Table S2: GBD 2023 Risk Factor Data Extraction and Selection Criteria; Table S3: World Population Prospects 2024 of the United Nations Department of Economic and Social Affairs, Population Division; Table S4: Age-specific population weights obtained from the SEER standard population dataset; Table S5: The disease burden of depressive disorders and their subtypes in the Asian; Table S6: Country-level EAPC of Incidence for Depressive Disorders (Both Sexes); Table S7: Country-level EAPC of Prevalence for Depressive Disorders (Both Sexes); Table S8: Country-level EAPC of DALYs for Depressive Disorders (Both Sexes); Table S9: Country-level EAPC of Incidence for MDD (Both Sexes); Table S10: Country-level EAPC of Prevalence for MDD (Both Sexes); Table S11: Country-level EAPC of DALYs for MDD (Both Sexes); Table S12: Country-level EAPC of Incidence for Dysthymia (Both Sexes); Table S13: Country-level EAPC of Prevalence for Dysthymia (Both Sexes); Table S14: Country-level EAPC of DALYs for Dysthymia (Both Sexes); Table S15: Incidence and incidence rates of depressive disorders and their subtypes (major depressive disorder and dysthymia) in Asia by age group and sex, 2023; Table S16: Prevalence and prevalence rates of depressive disorders and their subtypes (major depressive disorder and dysthymia) in Asia by age group and sex, 2023; Table S17: DALYs and DALYs rates of depressive disorders and their subtypes (major depressive disorder and dysthymia) in Asia by age group and sex, 2023; Table S18: Intimate partner violence; Table S19: Childhood sexual abuse; Table S20: Bullying victimization.

## Abbreviations

The following abbreviations are used in this manuscript:

MDD	Major depressive disorder
GBD	Global Burden of Disease Study
COVID-19	Coronavirus Disease 2019
DALYs	Disability-adjusted life years
YLLs	Years of life lost
YLDs	Years lived with disability
GHDx	Global Health Data Exchange
IPV	Intimate partner violence
CSA	Childhood sexual abuse
BV	Bullying victimization
ASR	Age-standardized rates
EAPC	Estimated annual percentage change
