# Supplementary material for: Temporal Trends in the Burden of Major Depressive Disorder and Dysthymia in Asia, 1990–2023: Findings from the Global Burden of Disease Study 2023
Source: Healthcare (Basel). 2026 Oct 22;14(21):3563. doi: 10.3390/healthcare14213563 (PMC13762932; doi:10.3390/healthcare14213563)
Supplement: Supplementary file 1 [file healthcare-14-03563-s001.zip › Table S1-S4.pdf]

Table S1. GBD 2023 Disease Burden Data Extraction and Selection Criteria

**Data source**	Global Burden of Disease Study 2023（GBD2023）
**Data source website**	https://vizhub.healthdata.org/gbd-results/
**GBD Estimate**	Cause of death or injury
**Measure**	Incidence、Prevalence、DALYs
**Metric**	Number, Rate
**Cause**	Depressive disorders;<br>Major depressive disorder;<br>Dysthymia.
**location**	Afghanistan, Armenia, Azerbaijan, Bahrain, Bangladesh, Bhutan, Brunei, Darussalam, Cambodia, China, Cyprus, Democratic People's Republic of Korea, Georgia, India, Indonesia, Iran (Islamic Republic of), Iraq, Israel, Japan, Jordan, Kazakhstan, Kuwait, Kyrgyzstan, Lao People's Democratic Republic, Lebanon, Malaysia, Maldives, Mongolia, Myanmar, Nepal, Oman, Pakistan, Palestine, Philippines, Qatar, Republic of Korea, Saudi Arabia, Singapore, Sri Lanka, Syrian Arab Republic, Taiwan (Province of China),Tajikistan, Thailand, Timor-Leste, Turkmenistan, Turkey, United Arab Emirates, Uzbekistan, Viet Nam, Yemen
**Age**	"0-4 years", "5-9 years", "10-14 years",<br>"15-19 years", "20-24 years", "25-29 years",<br>"30-34 years", "35-39 years", "40-44 years",<br>"45-49 years", "50-54 years", "55-59 years",<br>"60-64 years", "65-69 years", "70-74 years",<br>"75-79 years", "80-84 years", "85-89 years",<br>"90-94 years", "95+ years".
**Sex**	Male, Female, Both
**Year**	1990-2023

Table S2. GBD 2023 Risk Factor Data Extraction and Selection Criteria

**Data source**	Global Burden of Disease Study 2023（GBD2023）
**Data source website**	https://vizhub.healthdata.org/gbd-results/
**GBD Estimate**	Risk factors
**Metric**	Number, Rate, Percent
**Risk**	intimate partner violence,<br>sexual violence against children,<br>bullying victimization
**Cause**	Major depressive disorder
**location**	Afghanistan, Armenia, Azerbaijan, Bahrain, Bangladesh, Bhutan, Brunei, Darussalam, Cambodia, China, Cyprus, Democratic People's Republic of Korea, Georgia, India, Indonesia, Iran (Islamic Republic of), Iraq, Israel, Japan, Jordan, Kazakhstan, Kuwait, Kyrgyzstan, Lao People's Democratic Republic, Lebanon, Malaysia, Maldives, Mongolia, Myanmar, Nepal, Oman, Pakistan, Palestine, Philippines, Qatar, Republic of Korea, Saudi Arabia, Singapore, Sri Lanka, Syrian Arab Republic, Taiwan (Province of China),Tajikistan, Thailand, Timor-Leste, Turkmenistan, Turkey, United Arab Emirates, Uzbekistan, Viet Nam, Yemen
**Age**	"0-4 years", "5-9 years", "10-14 years",<br>"15-19 years", "20-24 years", "25-29 years",<br>"30-34 years", "35-39 years", "40-44 years",<br>"45-49 years", "50-54 years", "55-59 years",<br>"60-64 years", "65-69 years", "70-74 years",<br>"75-79 years", "80-84 years", "85-89 years",<br>"90-94 years", "95+ years".
**Sex**	Male, Female, Both
**Year**	1990, 2019, 2021, 2023

Table S3. World Population Prospects 2024 of the United Nations Department of Economic and Social Affairs, Population Division.

**Data source**	Global Burden of Disease Study 2023
**Data source website**	https://population.un.org/wpp/downloads
**Select the File Type**	Probabilistic Projections (PPP scenarios)
**Select the Major Topic/Special Groupings:**	Population
**Sub Group**	Probabilistic
**Files (Click to Download)**	Population by age - Female (XLSX)<br>Population by age - Male (XLSX)
**Description**	Population by - Female.<br>Population by - Male.

Data source: World (WHO 2000-2025) Standard - Standard Populations - SEER Datasets
Data source website: https://seer.cancer.gov/stdpopulations/world.who.html

Table S4. Age-specific population weights obtained from the SEER standard population dataset

Age Group	WHO World Standard (%)	Recalculation to add to 1,000,000	Rounded to Integers	Standard For SEER*Stat
0-4	8.86	88569.00085	88,569	88,569
5-9	8.69	86869.59564	86,870	86,870
10-14	8.6	85969.91053	85,970	85,970
15-19	8.47	84670.36537	84,670	84,670
20-24	8.22	82171.24007	82,171	82,171
25-29	7.93	79272.25471	79,272	79,272
30-34	7.61	76073.37432	76,073	76,073
35-39	7.15	71474.98376	71,475	71,475
40-44	6.59	65876.94307	65,877	65,877
45-49	6.04	60378.8674	60,379	60,379
50-54	5.37	53681.21158	53,681	53,681
55-59	4.55	45484.08057	45,484	45,484
60-64	3.72	37186.98456	37,187	37,187
65-69	2.96	29589.64362	29,590	29,590
70-74	2.21	22092.26771	22,092	22,092
75-79	1.52	15194.68186	15,195	15,195
80-84	0.91	9096.816114	9,097	9,097
85-89	0.44	4398.460539	4,398	4,398
90-94	0.15	1499.475184	1,499	1,500*
95-99	0.04	399.860049	400	400
100+	0.005	49.98250612	50	50
Total	100.035	1000000	999,999	1,000,000

* The Standard in SEER*Stat for the 90-94 age group needed to be increased by one so the total would sum to 1,000,000. That group was selected because it was the number closest to rounding up that did not.
